# A Precise Drunk Driving Detection Using Weighted Kernel Based on Electrocardiogram

**DOI:** 10.3390/s16050659

**Published:** 2016-05-09

**Authors:** Chung Kit Wu, Kim Fung Tsang, Hao Ran Chi, Faan Hei Hung

**Affiliations:** Department of Electronic Engineering, City University of Hong Kong, Hong Kong, China; chungkwu4-c@my.cityu.edu.hk (C.K.W.); haoranchi2-c@my.cityu.edu.hk (H.R.C.); fhhung4-c@my.cityu.edu.hk (F.H.H.)

**Keywords:** drunk driving detection, electrocardiogram, weighted kernel, feature extraction

## Abstract

Globally, 1.2 million people die and 50 million people are injured annually due to traffic accidents. These traffic accidents cost $500 billion dollars. Drunk drivers are found in 40% of the traffic crashes. Existing drunk driving detection (DDD) systems do not provide accurate detection and pre-warning concurrently. Electrocardiogram (ECG) is a proven biosignal that accurately and simultaneously reflects human’s biological status. In this letter, a classifier for DDD based on ECG is investigated in an attempt to reduce traffic accidents caused by drunk drivers. At this point, it appears that there is no known research or literature found on ECG classifier for DDD. To identify drunk syndromes, the ECG signals from drunk drivers are studied and analyzed. As such, a precise ECG-based DDD (ECG-DDD) using a weighted kernel is developed. From the measurements, 10 key features of ECG signals were identified. To incorporate the important features, the feature vectors are weighted in the customization of kernel functions. Four commonly adopted kernel functions are studied. Results reveal that weighted feature vectors improve the accuracy by 11% compared to the computation using the prime kernel. Evaluation shows that ECG-DDD improved the accuracy by 8% to 18% compared to prevailing methods.

## 1. Introduction

Drunk driving detection (DDD) is an efficient way to reduce the tragedies and the expenditures due to traffic accidents. In each year, more than 1.2 million people die and more than 50 million people are injured due to traffic accidents [1]. The expenditures on these traffic accidents account for 1% to 3% of the world’s GDP, and are more than $500 billion dollars [2]. The World Health Organization (WHO) cautioned that traffic accidents will become the fifth leading cause of death [1]. Among all traffic crashes, about 40% of the crashes are due to drunk driving which also counts about 22% of the traffic injury costs [3]. WHO concludes that 70% of the world’s population can be protected if strong restrictions are imposed on drunk drivers [4]. The development of DDD is vital because it will help to reduce traffic accidents and thus reduce expenditure accordingly.

DDD provides an early protection to both drivers and pedestrians by alerting the drunk drivers when the drunk status is detected. DDD can be categorized into three (3) types: direct detection (which is also known as blood alcohol test) [5], drivers’ behavior-based detection [6,7] and biosignal-based detections [8]. The first two types cannot meet the requirements of pre-warning, fully automated and high accuracy simultaneously while the third type suffices. In [8], the plethysmogram signal, which measures changes in an organ’s volume and thus resulting in a fluctuation in blood or air content, was used. However, this detection is time consuming and the accuracy has not been specified. In addition to plethysmogram, other biosignals such as Electrocardiogram (ECG) and Electroencephalography (EEG) may also accurately reflect the human’s status. By virtue of the measurement stability character [9], it will be shown that the ECG has a higher robustness. Moreover, it will be shown that the ECG signals exhibit more robust stability (>97%) than EEG signals (~85%).

The ECG signal, mainly characterized by P, Q, R, S, and T wave, is a proven signal to indicate intrinsic human status as it measures the electrical activities of the heart which is closely related to a human’s status [10]. An ECG-based DDD (ECG-DDD) using support vector machine (SVM) is proposed in this letter. It is pointed out that there is no related work on ECG-DDD and the proposed method is the first of its kind. In the design, two classes, namely normal and drunk, will be classified by an efficient machine learning algorithm, SVM. The ECG datasets of both normal and drunk are collected from the volunteers. After the ECG preprocessing is conducted, 10 indicative features will be extracted from the datasets. These features include the means and variances of P, R, S waves, R-R intervals, P-wave maximal duration (Pmax) and P-wave dispersion (Pd). In order to improve the accuracy, the feature vectors are then weighted by incorporating the important features before the kernel is customized. The weighted feature vectors are then applied to kernel functions. Four commonly adopted kernels namely linear, quadratic, polynomial and radial basis kernel [11,12] are evaluated and analyzed. These four kernel functions are used to design the classifiers individually and the classifier with the highest accuracy will be selected. The result demonstrates that the accuracy of ECG-DDD is improved by 8% to 18% compared to prevailing methods. The accuracy, sensitivity and specificity of ECG-DDD are 88%, 88% and 87% respectively. This letter is structured as follows. In Section 2, the design of ECG-DDD is illustrated. The results and evaluations are presented in Section 3 and the conclusion is drawn in Section 4.

## 2. Design of ECG-Based DDD (ECG-DDD)

**Figure 1:**
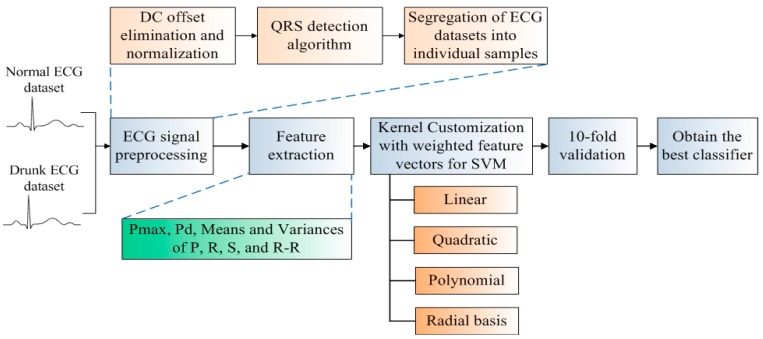
Classifier development flow of electrocardiogram drunk driving detection (ECG-DDD).

The classifier development flow of ECG-DDD is illustrated in Figure 1. Firstly, two datasets, the normal ECG signals and drunk ECG signals are collected from 50 volunteers for the development of the ECG-DDD classifier. ECG preprocessing is then applied for noise suppression and ECG signal segregation. An ECG signal is segregated into a number of samples and each sample represents the heart activity within one heartbeat. To identify indicative characteristics of “drunk”, key features including Pmax, Pd, means and variances of P, R, S waves and R-R intervals are extracted. The key feature developed in this investigation is that the feature vectors will be weighted to incorporate the important features during the kernel customization. The weighted features are then evaluated and analyzed in four commonly used kernel functions (linear, quadratic, polynomial and radial basis) for the development of classifiers. Hence, there are in total eight different kernel-based classifiers. To train the classifiers for performance evaluation, a well-known and convincing 10-fold cross validation is conducted [13]. The overall accuracy, sensitivity and specificity are evaluated and analyzed at each fold. Finally, the best classifier with the highest accuracy is selected.

### 2.1. ECG Datasets and Samples

**Figure 2:**
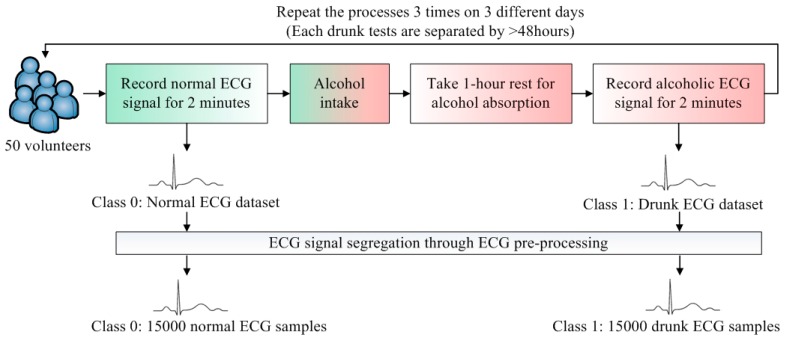
Flow of ECG data collection.

To develop a DDD classifier, two classes, Class 0 and Class 1, are defined. Class 0 represents the normal cases and Class 1 is the drunk cases. The flow of ECG data collection is illustrated in Figure 2. The datasets of Class 0 and Class 1 are obtained from 50 volunteers. To render the collected data realistic, the volunteers were sitting on the seat and held the steering wheel with two hands. Since the design of the ECG electrode is not the main focus in this paper, the minimum motion artifact is preferred during the data collection. The volunteers were asked to stay stationary and the standard wet ECG electrodes are placed on the chest. This placement can reduce the motion artifacts as the electrodes attached on the chest are less likely to be affected. 

For each volunteer, the normal ECG signal was recorded for 2 min. The relationship between the amount of alcohol intake and blood alcohol concentration (BAC) is related to several parameters. The Widmark formula is widely adopted to estimate BAC [14]. As such, the required amounts of alcohol intakes of all volunteers were estimated by the Widmark formula in order to reach BAC > 0.02% (0.02% BAC is the maximum legal levels of driving in China and Sweden [4]), the volunteer drank the estimated amount of alcohol and took a rest for 1 h. The 1-h rest facilitated the alcohol to be absorbed into human’s bloodstream [15]. After “BAC > 0.02%” is reached and confirmed by a breath alcohol analyzer, the drunk ECG signal was recorded for 2 min. This data collection was repeated 3 times. To suppress the effect of remaining alcohol in the bloodstream, the data collection was conducted on 3 different days. Each drunk test is separated by >48 h. 

The ECG samples with one heart beat duration are further segmented from two ECG datasets. The average heartbeat rate of Class 0 samples is about 71. To avoid the measurement instability at the beginning, the first 30 s of the recorded ECG signals are ignored. Then, the number of Class 0 samples is 71 × 50 × 3 × 1.5 = 15,975. To eliminate the corrupted samples (due to movement or friction of electrodes), the total number of Class 0 samples is 15,000. The Class 1 samples are more than Class 0 samples because of the faster average heartbeat rate in Class 1. It is concluded that if the sample sizes of the classes are unequal, the SVM classifier will be biased [16]. Hence, equal datasets of all classes are preferred. As such, a total of 15,000 samples of Class 1 was randomly selected in order to train the classifier fairly. The rest of the samples of Class 1 (apart from those 15,000 random selected samples) were not used in the following training and validation processes. There were 30,000 samples of these two classes which were sufficient for the training of SVM.

### 2.2. Preprocessing of ECG Signal

Preprocessing of ECG signal is required to suppress noises and segregate the ECG datasets into individual samples. The first step is to perform DC offset elimination and normalization on raw ECG signals in order to avoid voltage imbalance and thus render a useful and convenient representation. Practically, the R wave, normally the highest in amplitude, is selected to detect two sequence ECG samples as the P wave and the T wave are highly sensitive to noise. Meanwhile, the QRS detection algorithm is commonly and widely adopted and was utilized in the pre-processing [17]. The flow of the pre-processing is illustrated as follows:

1. Bandpass filter: During ECG signal segregation, the high energy components are utilized to distinguish two successive ECG samples because they are more likely to be identified. The energy of QRS components are relatively high and their energy spectra are concentrated within 5 Hz to 15 Hz [17]. The role of the bandpass filter is to filter out other unnecessary frequencies outside the range of QRS components.
2. Differentiation: The turning points of Q, R, and S waves can be estimated by the changes of their slopes. The peak positions and values of Q, R, and S can consequently be obtained from the estimated turning points. Hence, the slopes of Q, R, and S waves are determined at this stage. The peak-to-peak values of QRS can be computed by the five-point derivative filter.
3. Squaring and moving window integration: Signal squaring is a process that turns all data points positive and amplifies them for further processing. Practically, QRS cannot be determined by utilizing the slope of R alone because this slope can be different in many abnormal cases. Hence, moving window integration will be implemented to sort out more parameters such as QS interval. Extracted parameters will be used to determine QRS together with the slope of R.

It is found that the ECG samples can be segregated with 99% accuracy using QRS detection algorithm. By applying the relevant thresholds and peaks, the durations of P, Q, R, S, T can be determined [18].

### 2.3. Feature Extraction for SVM

Feature extraction is the most key component in the design of the classifier. Features are commonly found in most situations and various characteristics are demonstrated under various situations [19]. In [15], it was concluded that about 8% variation in the ECG characteristics could be considered as a significant change. By comparing the normal ECG signals and drunk ECG signals, the prominent variations (≥8%) of ECG characteristics between normal cases and drunk cases were obtained.

The prominent variations (variation in peak values, intervals, and durations of the waves) in the ECG characteristics between normal ECG signals and drunk ECG signals are analyzed from the collected datasets and summarized in Table 1.

**Figure 3:**
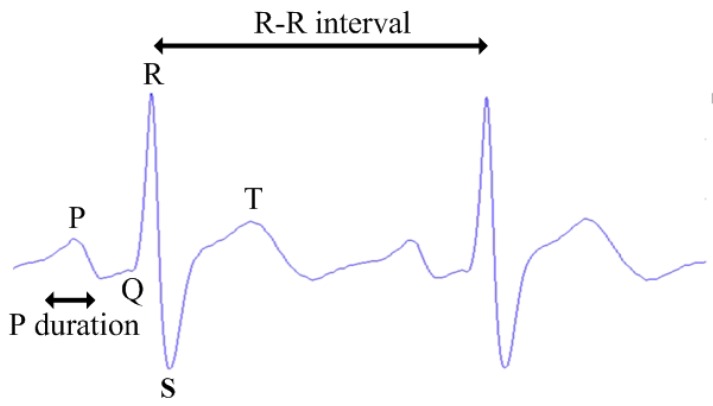
ECG characteristics.

It is noted that Pmax refers to the maximum duration of P wave and Pd refers to the difference between the maximum and minimum values of P wave duration [15]. The ECG characteristics with prominent variations in Table 1, as illustrated in Figure 3, fulfill the definition of feature [19] that they are commonly found in normal and drunk cases and demonstrate the differences between two cases. Therefore, to efficiently construct the feature vector, the following features are considered: the means and variances of P, R, S waves and R-R intervals together with Pmax and Pd. Thus, there are a total of 10 features in the feature vector.

### 2.4. Kernel for SVM

*K(x_i_*, *x_j_)* represents the kernel function for mapping the data to a feature space having higher dimension. There are four typical kernel functions [11,12]:

(1) $$\text{Linear kernel}\;K(x_{i},x_{j})=x_{i}\cdot x_{j}$$

(2) $$\text{Quadratic kernel}\;K(x_{i},x_{j})={\left(x_{i}\cdot x_{j}+1\right)}^{2}$$

(3) $$\text{Third order polynomial kernel}\;K(x_{i},x_{j})={\left(x_{i}\cdot x_{j}+1\right)}^{3}$$

(4) $$\text{Radial basis kernel}\;K(x_{i},x_{j})=\text{exp}(-\gamma ||x_{i}\cdot x_{j}|{|}^{2})\;\text{with}\;\gamma =1/2\sigma^{2}$$

where *x* refers to the feature vector.

The first kernel function is linear and the other three kernel functions are non-linear. The linear kernel is the simplest method because the hyperplane of separating two classes is a straight line. However, if the extracted features are non-linear, the accuracy of the linear kernel will be seriously degraded. The other three types are non-linear kernel functions. The polynomial kernel achieves good generalization but its learning capacity is relatively low [12]. Low learning capacity is usually due to the fact that the training data exceeds the dimensionality of feature space and subsequently results in a poor separating hyperplane [20]. The quadratic kernel and third order polynomial kernel both belong to the polynomial kernel but they are different in the degrees of fitting (Quadratic is 2 and third order polynomial is 3). The generalization of the polynomial kernel will degrade if the degree is high. This is also known as the over-fitting problem. Hence, the degree within 3 is recommended [12]. On the other hand, the Radial basis kernel has high learning capacity but it is relatively low in generalization [12]. The cross validation will be performed to select the best kernel for DDD.

However, the kernel functions cannot identify the significance of the features by themselves [21]. This is one of the limitations of the accuracy of the classifier. Hence, weighting is incorporated to the feature vectors in order to inherit the important features. In [21], the weighting concept was utilized to sort out the necessary features. The features were selected if its weighting was higher than a certain threshold. The method is suitable to the classifier with a large amount of extracted features. The dataset in [21] contains about 346 to 699 instances and the instances were sorted out to 7 to 10 features. Therefore, the weighting concept in [21] concerned the selection of features. In this investigation, the features are defined by analyzing the variations of the ECG signal between normal cases and drunk cases. Instead of sorting out the features, the weighting concept in this work is to assign the heavier weightings. The extracted feature will first undergo correlation. The features’ relationships between the normal cases and drunk cases are obtained and reflected in the resultant correlation coefficients. The larger absolute value in the normalized correlation coefficient indicates the higher correlated relationship. This means that the association of the classifier’s input and output will be more likely to be affected by the highly correlated features. The magnitude of the correlation coefficient will determine the level of importance of the feature. Hence, to improve the accuracy of the classifier, the heavier weightings are then assigned to the highly correlated features and *vice versa*. The weighted kernels *K_c_* (*x_i_*, *x_j_*) will be formulated as:

(5) $$K_{c}(x_{i},x_{j})=\sum_{p}c_{p}K_{p}(x_{i}^{p},x_{j}^{p})$$

where *c_p_* is the weighting of the corresponding feature *p*. 

The hyperplanes are to separate the classes so that no data points will exist between them. The distance between the data points is also defined as margin, is preferred to be as far as possible. Hence, the margin between two Classes (Class 0 and Class 1) should be maximized. To deal with the maximizing margin problem, the weighting *c_p_* of kernel *K_c_* is customized and the optimization function is formulated as:

(6) $$\begin{array}{l} \tilde{D}(\alpha ,c)=\arg\underset{\alpha }{\max}\{\sum_{i=1}^{N}\alpha_{i}-\frac{1}{2}\sum_{i=1}^{N}\sum_{j=1}^{N}\alpha_{i}\alpha_{j}b_{i}b_{j}K_{c}(x_{i},x_{j})\}\\ \text{s. t.}\{\begin{array}{c} \alpha_{i}\geq 0\\ \begin{array}{c} \sum_{i=1}^{N}\alpha_{i}b_{i}=0\\ \sum_{p}c_{p}=1 \end{array} \end{array}\begin{array}{c} \forall i=1,...,N \end{array} \end{array}$$

where *α* is known as Lagrange multiplier, *N* is the total number of training data points, *b* is {1,−1} and it is also known as the class label of the input data. 

## 3. Testing Results and Discussion

A 10-fold cross validation is a convincing choice for training and testing the classifier [13]. First, all ECG samples are divided into 10 groups randomly. At each fold, among 10 groups, 9 groups (90%) are used to train the classifier and the remaining 1 group (10%) is used to validate the classifier. After one fold validation is completed, at the next fold, the previous validation group becomes the training group and one of the previous training groups act as validation group. In particular, once the group is utilized to validate, this group will not act as the validation group in the rest of the folds. The group shifting repeats 9 times and hence all groups (10 out of 10) can be validated. The whole process is considered as 10 fold validation. In each fold, the accuracy, sensitivity and specificity are recorded.

The performance of the classifier is determined by the following parameters: accuracy (*Acc*), sensitivity (*Se*) and specificity (*Sp*). *ACC* measures the ratio of cases being classified correctly to the total cases. *Se* measures the ratio of drunk drivers that the test is positive to the total drunk cases. *Sp* is the ratio of normal drivers being classified correctly to the total normal cases. The parameters can be computed as [22,23]:

(7) Acc=(Tp+Tn)/(Tn+Tp+Fn+Fp)

(8) Se=Tp/(Tp+Fn)

(9) Sp=Tn/(Tn+Fp)

*Tp* and *Tn* represents the number of true positives and true negatives respectively. In this case, *Tp* is the drunk driver being classified correctly and *Tn* is the normal driver being classified correctly. *Fp* and *Fn* denotes the number of false positives and false negatives, respectively. The normal driver being classified to drunk driver is considered as *Fp* and the drunk driver being classified to normal driver is regarded as *Fn*.

The experimental data are processed by using the kernels in Table 2: Linear, Weighted Linear, Quadratic, Weighted Quadratic, Third order polynomial, Weighted Third order polynomial, Radial basis, and Weighted Radial basis. The *Acc*, *Se* and *Sp* are recorded at each fold during validation. The averaged values of *Acc*, *Se* and *Sp* of various kernels K1 to K4 are listed and compared as shown in Table 2.

The results show that K3a, the third order polynomial kernel based classifier achieves the highest *Acc* of 76% among the four (4) prime kernel functions. The linear kernel function yields the worst performance and it is caused by the non-linear drunk detection. Besides, the weighting concept improves the accuracy by 6% to 12%, showing that the weighted features render a higher accuracy. Among all weighted kernels, K3b, the weighted third order polynomial kernel based classifier gives the best performance, namely *ACC* of 87.52%, *Se* of 88.32%, and *Sp* of 86.71%. This kernel function is then adopted to design the classifier.

The ECG-DDD is compared to other literatures and listed in Table 3.

It is noted that the accuracies of DDD in [5,8] have not been specified and hence comparisons cannot be made. In this analysis, the results show that the ECG-DDD achieves 8% to 18% improvements on the detection accuracy. Besides, for drivers’ behavior-based DDD, it measures the driver’s behavior. The drawback is that no decision can be made before the driver’s behavior has greatly deviated from a normal situation. This leads to the serious delay of detection. The biosignal-based DDD measures the intrinsic human’s status and thus real-time detection and response can be provided. Moreover, modern wearables show that ECG sensors are ready to be installed. These facilitate the practical implementation of ECG-DDD.

Practically, it is preferred that the ECG measurement can be performed in the most convenient way. It implies that the drivers do not wear any extra equipment and perform the ECG measurements. The most straightforward solutions are to embed the ECG sensors on steering wheel and/or seat and measure ECG signals from drivers’ hands and/or chests. To realize it, the ECG electrodes are installed on the steering wheel and the seat. The ECG measurement relies on the potential difference between the electrodes. The placement of the ECG electrodes determines the viewing angle of the heart activity and hence the ECG waveforms can be different under different placements of electrodes. The placements of ECG electrodes should be consistent during data collection and practical implementation. At this point, it is known that there are two related projects carrying by two well-known automotive companies, namely Toyota and Ford [24,25]. The Toyota project embeds the ECG electrodes on the steering wheel which is a kind of direct contact measurement. This requires the driver to hold the steering wheel for a long time and usually deals with motion artifacts. On the other hand, the Ford project installs the ECG electrodes on the driver's seat and it is contactless ECG measurement. As such, it is required to deal with the motion artifacts and cloth thickness and the driver is possibly required to make as close contact with the seat as is possible.

## 4. Conclusions

Drunk driving detection (DDD) is an important and efficient way to reduce the tragedies due to drunk drivers. Electrocardiogram (ECG) is a reliable signal to indicate the status of humans. It appears that there is no known research or literature found on DDD using ECG. Hence, an ECG-based DDD (ECG-DDD) has been proposed using support vector machine (SVM). In the feature extraction, 10 prominent features are extracted from ECG signal based on the studies of the collected ECG data. The important features are further weighted. Four commonly adopted kernel functions are considered with the 10 extracted weighted features. It is evaluated and analyzed that the third order polynomial kernel achieves the highest accuracy. The results show that the weighted features concept improves the accuracy by 11% compared to the prime kernel. Also, the proposed work has improved the accuracy by 8% to 18% compared to prevailing methods. The accuracy, sensitivity and specificity of the proposed DDD are 87.52%, 88.32% and 86.71% respectively. Hence, the proposed work has shown that ECG-DDD is a robust and precise algorithm for DDD.

## Figures and Tables

**Table 1:** The variations of ECG characteristics between normal cases and drunk cases.

Characteristics	Variations (Averaged)
P wave peak value	−11.21%
R wave peak value	+19.54%
S wave peak value	+8.14%
R-R interval	−8.43%
P-wave maximum duration (Pmax)	+9.07%
P-wave dispersion (Pd)	+23.77%

**Table 2:** Comparison of typical kernel based classifier.

Kernel Types		*Acc*	*Se*	*Sp*
K1a	Linear	62.83%	60.34%	65.32%
K1b	Weighted Linear	69.04%	67.86%	70.22%
K2a	Quadratic	66.17%	67.07%	65.26%
K2b	Weighted Quadratic	75.61%	73.27%	77.95%
K3a	Third order polynomial	76.39%	77.17%	75.60%
K3b	Weighted Third order polynomial	87.52%	88.32%	86.71%
K4a	Radial basis	69.43%	68.75%	70.12%
K4b	Weighted Radial basis	81.76%	81.01%	82.49%

**Table 3:** Comparison of DDD.

Algorithms		*Acc*	*Se*	*Sp*
[6]	Drivers’ behavior-based: SVM	70%	75%	66%
[7]	Drivers’ behavior-based: Changes in acceleration	80%	NA	NA
Proposed work (ECG-DDD)	Biosignal based: SVM with weighted third order polynomial kernel	87.52%	88.32%	86.71%
